# Comparative Study of Complete Blood Count Between High-Altitude and Sea-Level Residents in West Saudi Arabia

**DOI:** 10.7759/cureus.44889

**Published:** 2023-09-08

**Authors:** Siraj B Alharthi, Ijtihed Kilani, Hawazen S Solaimani, Ahmed Y Salami, Nojood A Althubaity, Naif M Alosaimi, Abdullah S Alsulaiman, Mohamed H Zainy, Muhammad A Qureshi, Mohamed M Ahmed

**Affiliations:** 1 Biological Sciences Department, King Abdulaziz University, Taif, SAU; 2 Science Department, Shorouq Al Mamlakah International School, Taif, SAU; 3 Hematology Laboratory, King Fahad Armed Forces Hospital, Jeddah, SAU; 4 Hematology Department, Al Hada Armed Forces Hospital, Taif, SAU; 5 Immunology Department, Al Hada Armed Forces Hospital, Taif, SAU; 6 Molecular Diagnostic Unit, Al Hada Armed Forces Hospital, Taif, SAU; 7 Biological Sciences Department, King Abdulaziz University, Jeddah, SAU; 8 Clinical Laboratory Medicine Department, Al Hada Armed Forces Hospital, Taif, SAU; 9 City for Scientific Research and Technological Applications, Genetic Engineering and Biotechnology Research Institute (GEBRI), Alexandria, EGY

**Keywords:** sea level, complete blood count, effect of high altitude, cbc, hematology

## Abstract

The reduction in oxygen partial pressure at high altitudes leads to diminished oxygen saturation in the arteries, stimulating erythropoietin production and erythropoiesis to restore appropriate oxygenation. While many studies have explored acclimatization to high altitude and its effects on complete blood count (CBC) parameters, our research uniquely examined both male and female healthy individuals, emphasizing the novelty of gender-specific observations. We analyzed 1,160 individuals in Taif (Al Hada), east Saudi Arabia, a high-altitude region, and compared them to 1,044 counterparts in Jeddah, at sea level. Our results revealed significant variations in CBC parameters, including white blood count, red blood count, hemoglobin, hematocrit, platelets, neutrophils, lymphocytes, monocytes, eosinophils, and basophils, reflecting the body's hypoxic response. These variations were observed in both genders, with specific differences noted between males and females. For example, NEU (neutrophils), representing the absolute count of a type of white blood cell essential in the immune system's defense, showed significant variations for males. The male results show that the variation in males between the sea level and high altitudes indicated significant p-values for all CBC parameters except NEU between at sea level (Jeddah city), whose p-value was 0.8696, and at high altitude (Taif city, Al Hada). In contrast, MONO (monocytes), another type of white blood cell involved in immune response, and RBC (red blood cells), responsible for oxygen transport, were mentioned but did not show significant variations for females. The full results for females showed significant results (P<0.0001) for BASO, HCT, HGB, MCH, MCHC, MPV, PLT, RDW, and WBC between the sea-level altitude and high altitude for females. Also, EOS and LYM showed significant P-values of 0.0002 and 0.0001, respectively, while MONO, NEU, and RBC indicated no significance between the sea-level altitude and high altitude for females. The p-values of MONO, NEU, and RBC, respectively, were 0.1907, 0.1259, and 0.0677. The results for both genders combined showed significant variations of all CBC parameters (P<0.0001) between the sea-level altitude and high altitude except for MONO, NEU, and RBC, which were not significant for both males and females, with p-values of 0.1589, 0.2911, and 0.0595, respectively. All unhealthy individuals were excluded from the study with any condition that would cause significant changes in CBC parameters and would skew the results, ensuring a focus on physiological adaptations in healthy subjects. By comparing healthy individuals and examining each gender separately, this study contributes valuable insights into high-altitude acclimatization, enhancing our understanding of physiological adaptations and potentially guiding health management in such environments within the normal range.

## Introduction

High altitude (HA), defined as elevations ranging from 2000 to 5500 meters above sea level, presents a unique physiological challenge characterized by reduced oxygen availability and atmospheric pressure. These conditions culminate in hypobaric hypoxia, a state that triggers a series of adaptive hematological responses within the human body [1]. The physiological adaptations to high altitude have been the subject of extensive research, with particular emphasis on the hematological changes that occur in response to chronic hypoxia [2].

The complete blood count (CBC) test serves as a critical diagnostic tool to elucidate these adaptations [3]. At high altitudes, CBC parameters undergo significant alterations, reflecting the body's response to hypoxic conditions. An increase in red blood count (RBC) and hemoglobin (Hgb) levels is observed, driven by erythropoietin (EPO) production, enhancing oxygen-carrying capacity [4]. Elevated hematocrit levels optimize the oxygen delivery to tissues and lead to increased blood viscosity. Changes in white blood count (WBC) and its subtypes may reflect immunological adaptations to the hypoxic environment while the response in platelet counts at high altitudes remains complex and multifaceted.

A significant aspect of adaptation to high altitude is the increase in hemoglobin levels, which requires high amounts of additional iron supplied from the diet [5]. Long-term adaptation to high altitude enables healthy individuals to maintain their iron stores within the physiological range despite elevated requirements for erythropoiesis. However, in vulnerable populations with increased iron demand, iron stores are less likely to be replenished quickly when living at high altitudes [5].

This research aims to explore these hematological adaptations by examining CBC parameters in a cohort of individuals residing in Taif (Al Hada), West Saudi Arabia, a high-altitude region that is 2177 meters above sea level, and comparing them to counterparts in Jeddah, at sea level. The study includes both male and female participants and the parameters under investigation include BASO (basophils; G/L or %), EOS (eosinophils; G/L or %), HCT (hematocrit; L/L), HGB (hemoglobin; g/L), LYM (lymphocytes; G/L or %), MCH (mean corpuscular hemoglobin; pg), MCHC (mean corpuscular hemoglobin concentration; g/dL), MCV (mean corpuscular volume; fL), MONO (monocytes; G/L or %), MPV (mean platelet volume; fL), NEU (neutrophils; G/L or %), PLT (platelets; x10^9/L), RBC (red blood cells; x10^12/L), RDW (red cell distribution width (CV%), WBC (white blood cells; x10^9/L). By focusing on these specific CBC parameters, this study seeks to contribute novel insights into the physiological adaptations to high altitude, adding valuable information to the field of high-altitude biology and medicine.

The variability in these parameters has spurred interest in understanding the underlying genetic and immunological factors contributing to these changes [6]. The genetic background of an individual, together with epigenetic modifications, may determine the development of moderate or excessive erythrocytosis in response to different stimuli, epigenetic processes, and physiological events throughout the lifespan [4].

## Materials and methods

Aim of the study

The objective of this rigorous research endeavor was to delve deeply into the potential variations observed in CBC parameters among individuals residing in two geographically and altitudinally distinct regions: Jeddah City, situated at sea level, and Taif City, perched atop Al Hada Mountain, representing a high-altitude environment. By undertaking this study, the intention was to provide a granular understanding of how altitude, a significant environmental factor, might exert influence on specific hematological parameters, thereby offering insights into broader physiological implications. Understanding if the genetic background of an individual, together with epigenetic modifications, may determine the development of moderate or excessive erythrocytosis in response to different stimuli, epigenetic processes, and physiological events throughout the lifespan.

Study area/setting

This research was meticulously designed and executed across two strategically chosen western regions within the Kingdom of Saudi Arabia, each offering a unique altitudinal perspective: Jeddah City: Jeddah represents a sea-level environment. Its unique coastal climate, coupled with its urbanized setting, offers a multifaceted environment for the study.

Taif City (Al Hada Mountain): Elevated at a significant 2177 meters above sea level, Taif’s location within the Al Hada Mountain range introduces a set of variables that are inherent to mountainous, high-altitude regions, making it an ideal setting for this research.

Data collection methods

Source of Data

The foundational data for this research was meticulously sourced from a vast repository of retrospective lab records maintained by renowned Armed Forces hospitals within the regions of interest. King Fahad Armed Forces (sea level) and Al Hada Armed Forces (high altitude) hospitals. *Instrumentation*

For the purpose of this research, the Alinity hq analyzer (Abbott Laboratories, Abbott Park, Illinois) was employed. The Alinity hq is a state-of-the-art hematology diagnostic instrument known for its precision, accuracy, and reliability in analyzing blood samples. Its advanced technology ensures that the CBC parameters are measured with utmost accuracy, providing a robust foundation for the research's findings.

Inclusion Criteria

The study was discerning in its participant selection, focusing on individuals aged between 40 to 80 years. Their health and medical status were rigorously verified through an exhaustive review of medical files, detailed clinical histories, and other pertinent medical documentation. For thorough and reliable research results, race was taken into consideration when the study participants were selected, as it has been linked to a number of factors, including health and genetics, which are shared by the groups being studied. To limit the variables being compared, an age group of 40-80 was selected as the focus of the study. More than one technologist was involved in performing the test; however, the biases were minimized by having standardized specimen collection protocols and analyzing specimens on an automated analyzer as a part of a total lab automation unit without human handling in the preanalytical, analytical, and post-analytical phases of testing.

Exclusion Criteria

To maintain the integrity and purity of the data, participants who had undergone blood transfusions recently, individuals with known chronic illnesses, those on medications that could influence blood parameters, and anyone with a history of hematological disorders were systematically excluded. Furthermore, any participant deemed unhealthy based on their medical records or those with conditions that could potentially skew the results were also excluded from the study. Any condition that would cause significant changes in CBC parameters and would skew the results, including, but not limited to anemia, leukemia, polycythemia vera, thrombocytopenia, thrombocytosis, leukocytosis, leukopenia, neutropenia, lymphocytosis, hemolytic anemia, aplastic anemia, and infectious mononucleosis.

Data Handling, Processing, and Confidentiality

Utmost care was taken to de-identify all participant data, ensuring the removal of any personal identifiers. This anonymized data was then systematically organized into a structured Excel datasheet (Microsoft Corporation, Redmond, WA), primed for subsequent analysis. Post-extraction, the data were subjected to a thorough revision and coding process before being integrated into the statistical software GraphPad Prism 8.4.2 (679) (GraphPad Software, Boston, Massachusetts) for in-depth analysis. Furthermore, we included references ranges that are: EOS (0.000-0.500), HCT (0.36-0.46), HGB (120-180), LYM (1.00-4.40), MCH (27.0-32.0), MCHC (320-350), MCV (76.0-96.0), MONO (0.100-1.100), MPV (6.10-11.10), NEU (2.00-7.50), PLT (150-400), RBC (3.80-4.80), RDW (11.5-14.5), WBC (4.00-11.00), and BASO (0.00-0.100).

Study plan

Spanning a dedicated timeframe of three years (2021-2023), the research undertook a comprehensive analysis of a plethora of CBC parameters, including but not limited to BASO, EOS, HCT, HGB, and LYM. The data sets, both gender-specific and combined, were subjected to rigorous statistical scrutiny. The analytical process encompassed computing descriptive mean ± standard deviation and conducting an unpaired t-test with Welch's correction. This was done to discern any significant differences in CBC parameters between the two altitudinal regions.

Sample size

A total of 12, 438 people were initially screened for possible study subjects. After applying the inclusion and exclusion criteria, a total of 10,234 subjects were excluded from the study and 2,204 eligible subjects were included in the study. The selected people were screened for their health and medical status through an exhaustive review of medical files, detailed clinical histories, and other pertinent medical documentation. It was a non-randomized, retrospective review of medical records and data.

Ethical considerations

Adhering to the highest standards of academic research, the study protocol was crafted with a keen emphasis on ethical considerations. It received the esteemed approval from the ethical and research committee of King Fahad Armed Forces Hospital, as evidenced by Reference Ethical Number: REC 504. Every step, from data collection to analysis, was conducted with an unwavering commitment to upholding the principles of ethical research.

## Results

After applying age and lab exclusion criteria, we screened a total of 1,160 participants from Taif (Female: 411, Male: 749) and 1,044 participants from Jeddah (Female: 535, Male: 509). To minimize confounding variables, we matched the two groups based on several baseline characteristics, including the number of participants, age, and gender distribution. Specifically, the mean age of participants in Taif was 57 years while in Jeddah, it was 55 years. There is no notable difference between both groups. These characteristics were integral parts of our inclusion and exclusion criteria. Table 1 showcased significant variations of all CBC parameters (P<0.0001) between the sea-level altitude and high altitude except for MONO, NEU, and RBC, which were not significant for both males and females.

**Table 1:** Descriptive mean ± standard deviation and student t-test for males and females’ CBC parameters at sea level (Jeddah City) and high altitude (Taif City) Significant at 0.05*, 0.01**, 0.001***, 0.0001****. SD: Standard Deviation.

Parameter (Unit)	Taif		Jeddah		P-value
	N	Mean±SD	N	Mean±SD	
BASO (x10^9/L)	1160	0.038±0.026	1044	0.061±0.014	<0.0001****
EOS (x10^9/L)	1160	0.203±0.125	1044	0.188±0.091	<0.0001****
HCT (L/L)	1160	0.439±0.051	1044	0.415±0.520	<0.0001****
HGB (g/L)	1160	158.5±17.83	1044	136.4±13.85	<0.0001****
LYM (x10^9/L)	1160	2.266±0.783	1044	2.499±0.579	<0.0001****
MCH (pg)	1160	29.85±1.550	1044	28.60±3.510	<0.0001****
MCHC (L/L)	1160	330.5±9.489	1044	323.1±13.22	<0.0001****
MCV (fL)	1160	90.32±4.330	1044	87.96±8.212	<0.0001****
MONO (x10^9/L)	1160	0.587±0.180	1044	0.576±0.191	0.1589
MPV (fL)	1160	9.211±1.188	1044	10.61±0.903	<0.0001****
NEU (x10^9/L)	1160	3.606±1.413	1044	3.49±1.70	0.2911
PLT (x10^9/L)	1100	292.4±62.26	1044	256.6±63.97	<0.0001****
RBC (x10^9/L)	1160	4.864±0.601	1044	4.769±1.522	0.0595
RDW (CV%)	1160	13.04±0.843	1044	13.79±1.547	<0.0001****
WBC (x10^9/L)	1160	5.38±1.33	1044	6.77±1.77	<0.0001****

Table 2 indicated significant results (P<0.0001) for BASO, HCT, HGB, MCH, MCHC, MPV, PLT, RDW, and WBC between the sea-level altitude and high altitude for females. Also, EOS and LYM showed significant p-values of 0.0002 and 0.0001, respectively, while MONO, NEU, and RBC indicated no significance between the sea-level altitude and high altitude for females.

**Table 2:** Descriptive mean ± standard deviation and student t-test for females’ CBC parameters at sea level (Jeddah City) and high altitude (Taif City) Significant at 0.05*, 0.01**, 0.001***, 0.0001****. SD: Standard Deviation.

Parameter (Unit)	Taif		Jeddah		P-value
	N	Mean±SD	N	Mean±SD	
BASO (x10^9/L)	411	0.038±0.027	535	0.052±0.0.026	<0.0001****
EOS (x10^9/L)	411	0.186±0.133	535	0.231±0.071	0.0002***
HCT (L/L)	411	0.397±0.029	535	0.388±0.039	<0.0001****
HGB (g/L)	411	138.5±7.600	535	128.6±8.050	<0.0001****
LYM (x10^9/L)	411	2.284±0.816	535	2.49±0.79	<0.0001****
MCH (pg)	411	29.91±1.537	535	28.02±2.926	<0.0001****
MCHC (L/L)	411	329.3±9.335	535	321.2±8.636	<0.0001****
MCV (fL)	411	90.89±4.316	535	87.04±9.245	<0.0001****
MONO (x10^9/L)	411	0.544±0.164	535	0.573±0.160	0.1907
MPV (fL)	411	9.304±1.15	535	10.65±0.994	<0.0001****
NEU (x10^9/L)	411	3.504±1.386	535	3.43±1.15	0.1259
PLT (x10^9/L)	411	297.2±56.86	535	263.7±66.75	<0.0001****
RBC (x10^9/L)	411	4.375±0.350	535	4.539±2.034	0.0677
RDW (CV%)	411	12.97±0.837	535	14.05±1.771	<0.0001****
WBC (x10^9/L)	411	5.399±1.334	535	6.865±1.855	<0.0001****

As shown in Table 3, the variation in males between the sea level and high altitudes indicated significant p-values for all CBC parameters except NEU between sea level (Jeddah City) and high altitude (Taif City, Al Hada).

**Table 3:** Descriptive mean ± standard deviation and student t-test for males’ CBC parameters at sea level (Jeddah City) and high altitude (Taif City) Significant at 0.05*, 0.01**, 0.001***, 0.0001****. SD: Standard Deviation.

Parameter (Unit)	Taif		Jeddah		P-value
	N	Mean±SD	N	Mean±SD	
BASO (x10^9/L)	749	0.038±0.026	509	0.054±0.022	<0.0001****
EOS (x10^9/L)	749	0.213±0.120	509	0.235±0.145	0.0007***
HCT (L/L)	749	0.213±0.12	509	0.22±0.149	<0.0001****
HGB (g/L)	749	169.8±10.63	509	144.6±13.96	<0.0001****
LYM (x10^9/L)	749	2.255±0.765	509	2.53±0.58	0.0010***
MCH (pg)	749	29.81±1.558	509	29.2±3.957	0.0010**
MCHC (L/L)	749	331.1±9.517	509	325.0±16.52	<0.0001****
MCV (fL)	749	90.0±4.309	509	88.92±6.842	0.0017**
MONO (x10^9/L)	749	0.610±0.184	509	0.590±0.160	0.0398*
MPV (fL)	749	9.160±1.207	509	10.58±0.848	<0.0001****
NEU (x10^9/L)	749	3.662±1.425	509	3.43±1.16	0.8696
PLT (x10^9/L)	749	289.7±65.00	509	249.0±60.07	<0.0001****
RBC (x10^9/L)	749	5.132±0.538	509	5.011±0.539	<0.0001****
RDW (CV%)	749	13.07±0.845	509	13.51±1.211	<0.0001****
WBC (x10^9/L)	749	5.364±1.328	509	6.659±1.672	<0.0001****

**Figure 1:**
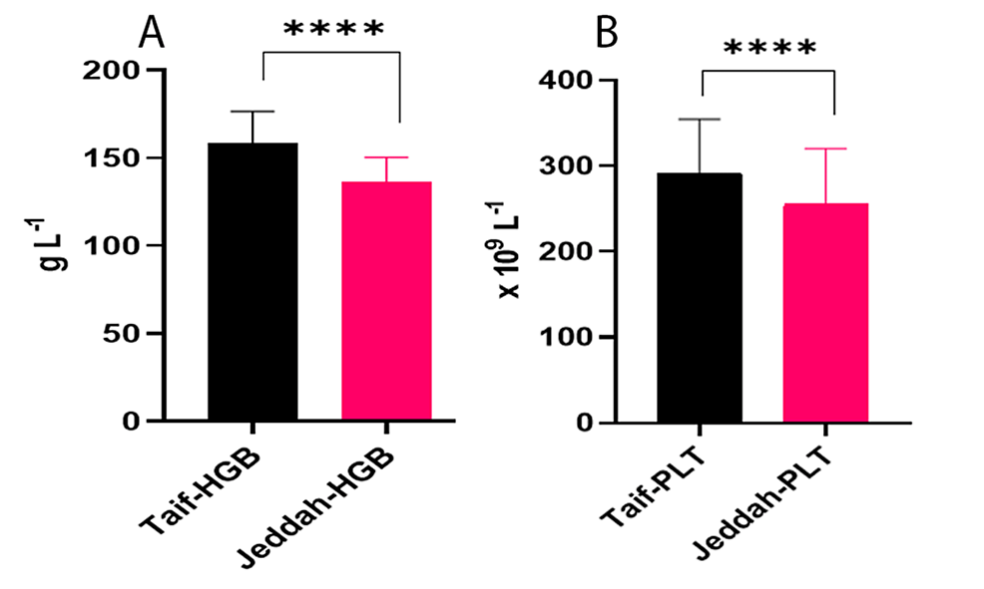
A: Statistical significance of unpaired t-test with Welch's correction analysis of the HGB parameters of males and females at sea level (Jeddah City) and high altitude (Taif City, Al Hada - 2177 m); B: Statistical significance of unpaired t-test with Welch's correction analysis of males and females’ blood PLT parameters at sea level (Jeddah City) and high altitude (Taif City, Al Hada)

As depicted in Figure 1, the analysis reveals distinct variations in the HGB and blood PLT parameters between males and females at different altitudes. Part A of the figure demonstrates significant differences in HGB levels for both genders when comparing the sea level of Jeddah city to the high altitude of Taif city Al Hada, which is situated at 2177 meters above sea level. Similarly, Part B of the figure highlights the statistical significance of blood PLT parameters for males and females under the same altitude conditions. The utilization of the unpaired t-test with Welch's correction further substantiates the findings, emphasizing the physiological impact of altitude on these blood parameters.

## Discussion

The present study conducted in Taif (Al Hada), West Saudi Arabia, one of the highest mountains in the region at over 2170 meters, and Jeddah, at sea level, provides a comprehensive and novel analysis of CBC parameters for both males and females, as well as their combined results. This unique approach reveals significant variations that reflect the body's hypoxic response and underscores the importance of gender-specific considerations [7].

For males, the study found significant variations in all CBC parameters except NEU (P=0.8696), as shown in Table 3. Specifically, the mean HGB for males was 169.8±10.63 in Taif and 144.6±13.96 in Jeddah (P<0.0001) while the mean PLT was 289.7±65.00 in Taif and 249.0±60.07 in Jeddah (P<0.0001) [8-11]. These findings highlight the distinct hematological adaptations in males in response to high altitude due to hypoxia [12].

For females, the results were equally revealing. Table 2 indicated significant results (P<0.0001) for BASO, HCT, HGB, MCH, MCHC, MPV, PLT [12], RDW, and WBC between the sea-level altitude and high altitude, while MONO, NEU, and RBC indicated no significance. The mean HGB for females was 138.5±7.600 in Taif and 128.6±8.050 in Jeddah (P<0.0001), and the mean PLT was 297.2±56.86 in Taif and 263.7±66.75 in Jeddah (P<0.0001) [11,12]. These results elucidate the specific variations in females, adding a new dimension to our understanding of high-altitude acclimatization.

The combined results for both males and females, as detailed in Table 1, further emphasize the significant variations in CBC parameters. The mean WBC, RBC, and HBG, as shown in Figure 1A (HGB) [10,11], HCT and PLT, as shown in Figure 1B, polys, LYM, MONO, EOS, and BASOS were found to vary significantly between the two altitudes, reflecting the body's hypoxic response [9]. Specifically, the parameters MONO (P=0.1589) and NEU (P=0.2911) were not significant while all other parameters showed significant variations (P<0.0001).

This comprehensive analysis of both genders and their combined results represents a novel contribution to the field, providing valuable insights that may guide future research and health management in high-altitude environments [10,12,13]. The results may suggest that we may need to change the references depending on males and females generally, and specifically in West Saudi Arabia, which may propose more epigenetic research in the future [14]. The novelty of this research lies in its focus on the differentiation between CBC within healthy individuals in all parameters, differences between males and females in all these parameters, and comparisons between males and females at sea level and higher altitudes [15].

Through the juxtaposition of healthy subjects and by conducting a meticulous examination of each gender both individually and in aggregate, this investigation furnishes an idiosyncratic viewpoint on the process of acclimatization at elevated altitudes [16]. This insight augments our comprehension of the physiological modifications that transpire under such conditions. The innovation of this scholarly inquiry resides in its concentrated emphasis on delineating the disparities in CBC among healthy participants across all measurable parameters. It further extends to discerning the distinctions between male and female subjects within these parameters and orchestrating comparative analyses between male and female responses at sea level and conventional altitudes [17]. The structure and focus of this research contribute to a nuanced understanding of the complex interplay between gender, health, and adaptation to different atmospheric pressures and oxygen concentrations. This innovative approach sets the study apart, offering a groundbreaking perspective on the complex interplay between genetics, epigenetics, and environmental factors in shaping the human body's response to hypoxia.

Limitations

The limitations of the study include non-randomized sampling and underrepresentation of the young adult population between 18 and 40 years.

## Conclusions

The present study meticulously analyzed CBC parameters among 2204 participants from two distinct geographical altitudes: Jeddah City (sea level) and Taif City (Al Hada, high altitude, 2177 m). Utilizing a comparative retrospective lab records review, the study revealed significant variations in most CBC parameters (P<0.0001) between high altitude and sea level, except for MONO, NEU, and RBC, which were not significant for both males and females. The findings corroborate hypothesis H0, indicating that there are indeed significant differences among CBC parameters due to high altitude. This research contributes valuable insights into the physiological adaptations to altitude and may have implications for medical practices in high-altitude regions. The robust statistical analysis and ethical considerations further strengthen the validity of the study, although future research may explore the underlying mechanisms of these variations and conduct bigger-scale research all around Saudi Arabia to analyze those values.
